# Predictors of surgical complications of nephrectomy for urolithiasis

**DOI:** 10.1590/S1677-5538.IBJU.2018.0246

**Published:** 2019

**Authors:** Alexandre Danilovic, Thiago Augusto Cunha Ferreira, Gilvan Vinícius de Azevedo Maia, Fabio Cesar Miranda Torricelli, Eduardo Mazzucchi, William Carlos Nahas, Miguel Srougi

**Affiliations:** 1 Divisão de Urologia do Hospital das Clínicas da Faculdade de Medicina da Universidade de São Paulo, São Paulo, SP, Brasil

**Keywords:** Nephrectomy, Urolithiasis, Postoperative Complications

## Abstract

**Objectives:**

Nephrectomy due to stone disease may be a challenging procedure owing to the presence of significant inflammation and infection, involving high complication rate. The objective of our study was to identify predictors for complications of nephrectomy for urolithiasis.

**Patients and Methods:**

A retrospective review of 149 consecutive patients > 18y submitted to simple nephrectomy for urolithiasis from January 2006 to July 2012 was performed. Clinical data, computed tomography findings and pathology report were analyzed. Postoperative complications were categorized based on Clavien - Dindo classification. Logistic multivariate regression models assessed the predictors for surgical complications of nephrectomy for urolithiasis.

**Results:**

Eighty-three (55.7%) patients were submitted to laparoscopic nephrectomy and 66 (44.2%) to open procedure. Conversion to open surgery was necessary in 19.2% (16 / 83). On univariable analysis, higher preoperative chronic kidney stage (p = 0.02), Charlson comorbidity index ≥ 2 (p = 0.04), higher ASA score (p = 0.001), urgency due to sepsis (p = 0.01), kidney size ≥ 12 cm (p = 0.006), renal and perirenal abscess (p = 0.004 and 0.002 respectively) and visceral adhesion (p = 0.04) were associated with Clavien - Dindo score > 1. On multivariate analysis, higher ASA score (p = 0.01), urgency due to sepsis (p = 0.03), kidney size ≥ 12 cm (p = 0.04) and preoperative abscess (p = 0.04) remained significantly associated with complications. End - stage renal disease with dialysis was needed post - operatively in 3.4% (5 / 144) of patients.

**Conclusions:**

We identified that higher ASA score, urgency due to sepsis, kidney size ≥ 12 cm and preoperative abscess were associated with Clavien - Dindo score > 1.

## INTRODUCTION

The incidence of upper urinary tract stones has increased worldwide (1). Despite the introduction of minimal invasive techniques in the treatment of kidney stones, like percutaneous nephrolithotomy, shock wave lithotripsy and ureteroscopy, there are still some patients who need nephrectomy. There are currently few indications for simple nephrectomy including renal poor function with recurrent infections, pain, abscess, fistulization and suspected malignant transformation.

Simple nephrectomy is the standard procedure for the removal of a non - functioning benign kidney and does not require excision of the adrenal or any adenopathies. However, nephrectomy due to urolithiasis may be a challenging procedure when significant inflammatory, fibrotic, and infectious component is present. In these cases, complication rates are reported to be even higher than those in nephrectomy due to renal tumors (2).

As far as we known, there are no studies analyzing predictors for complications of simple nephrectomy specifically due to kidney stones. Our aim was to identify predictors for surgical complications of nephrectomy for urolithiasis.

## PATIENTS AND METHODS

In this retrospective study, we reviewed the records of 149 consecutive patients submitted to simple nephrectomy for urolithiasis from January 2006 to July 2012. All of them had > 18 years of age. Nephrectomy was accomplished due to recurrent and / or serious renal infection based on obstructive or very large urolithiasis (in an urgent setting) or due to complete loss of renal function based on non - treated obstructive nephrolithiasis. Consent was obtained for all patients. The local Institutional review board approval of the study protocol was obtained. The clinical data, comorbidities, preoperative computed tomography findings (stone location and composition, kidney size, presence of abscess, adherence and fistula), side of the kidney affected, character of the surgery (elective or urgency due to sepsis), pathology report and complications were analyzed.

Pre - and postoperative renal function was assessed by the equation of the Modification Diet for Renal Disease (MDRD) (3) for estimated glomerular filtration rate and was staged according to the National Kidney Foundation. Separate renal function was estimated by preoperative technetium – 99 m dimercaptosuccinic acid (DMSA) renal scintigraphy. Comorbidity was evaluated by Charlson Comorbidity Index (CCI) (4) and American Society of Anesthesiologists (ASA) score (5). The Clavien - Dindo Classification System (6) was used to categorize the postoperative complications.

In elective procedures, prophylactic antibiotic therapy was guided by urine culture. In negative tests, a single dose of first - generation cephalosporin was administered. In cases of positive urine culture, patients received prior targeted treatment. In emergency procedures, patients received third generation cephalosporin at least 24 hours prior to surgery. Laparoscopic or open approach was selected depending on patient and surgeon preference. Laparoscopic nephrectomy was performed outside Gerota's fascia as previously reported (7).

Logistic multivariate regression models assessed the predictors for surgical complications. Primary endpoint was Clavien - Dindo score > 1. We performed a subanalysis involving postoperative dialysis. Statistical analyzes were conducted with the aid of SPSS Statistics v16.0 (SPSS for Windows, Version 16.0. Chicago, SPSS Inc.).

## RESULTS

All patient characteristics and perioperative outcomes are presented in Table-1. Eighty - three (55.7%) patients were submitted to laparoscopic nephrectomy and 66 (44.2%) patients to open procedure. Conversion to open surgery was necessary in 19.2% (16 / 83) of laparoscopic procedures. The main cause for conversion was inadequate exposure of renal hilum due to severe adhesion an inflammation, seen in all cases. Additional causes for conversion included excessive bleeding during the operation (6 / 37.5%) and large intestinal injury (3 / 18.8%). Blood transfusion rate was 9.3%. The general mean hospitalization period was 6.4 ± 8.4 days, and open, laparoscopic and converted group mean hospitalization period were 6.7 ± 8.8, 6.4 ± 8.4 and 6.2 ± 8.3 days consecutively.

**Table1:** Patient Characteristics and Perioperative Outcomes.

Patients		149
	Male	35 (23.4)
	Female	114 (76.6)
Age (year)		47.9±14.2
BMI mean/SD (kg/m^2^)		26.6±5.4
Prior Renal Surgery		60 (40.2)
Urgency (Sepsis)		32 (21.4)
Renal Size mean/OR, cm		11.7±3.8
**Stone location**		
	Staghorn	110 (73.8)
	Pelvic	18 (12)
	Ureteral	21 (14.0)
**Stone composition**		
	Struvite	64 (42.9)
	Calcium	60 (40.2)
	Cistine	2 (1.3)
Mixed		23 (15.4)
Left Kidney		72 (48.3)
MDRD mean/SD (mL/min/1.73m2)		70±27.9
Charlson mean/SD		1.2±1.8
Diabetes		21 (14.0)
Hypertension		67 (44.9)
ASA		
	I	43 (28.8)
	II	74 (49.6)
	III	28 (18.7)
	IV	4 (2.6)
DMSA scan in affected renal unit % (mean)		9.76
**Tomographic Findings**		
	Hydronephrosis	119 (79.8)
	Fat Changes	109 (73.1)
	Renal Abscess	53 (35.5)
	Perirenal Abscess	33 (22.1)
	Pararenal Abscess	22 (14.7)
	Adherence to the liver/spleen	47 (31.5)
	Adherence to the bowel	36 (24.1)
	Adherence to the muscle	38 (25.5)
Laparoscopic		83 (55.7)
Conversion		16/83 (19.2)
Hospital Stay (day)		6.4±8.4
**Pathology Report**		
	Xanthogranulomatous Pyelonephritis	48(32.2)
	Chronic pyelonephritis	43(28.8)
	Pyonephrosis	35(23.4)
	Kidney atrophy	19(12.7)
	Nephrocalcinosis	4(2.6)

**BMI =** Body Mass Index; MDRD = Modification of Diet in Renal Disease; **CCI =** Charlson Comorbidity Index; **ASA =** American Society of Anesthesiologists; **DMSA =** technetium-99m dimercaptosuccinic acid.

Clavien - Dindo score > 1 was reported in 28 (18.7%) patients (Table-2). Five patients had vascular injury, three involving vena cava, one involving iliac artery and one involving colonic artery. There were three cases of duodenal injury (two laparoscopic and one open surgery) and all of them were repaired during the procedure. One of them needed conversion to open access. Two pleural injuries occurred and were repaired with simple suture and temporary thoracic drainage. Open splenectomy at immediate postoperative period was performed in one case to treat a peritoneal bleeding due to spleen laceration. The median of postoperative estimated glomerular filtration rate based on MDRD equation was 64.7 and 61.3 mL / min / 1.73 m^2^ in Clavien ≤ 1 and Clavien > 1 groups (p = 0.52). Eleven patients were on stage V of chronic kidney disease and five of them on dialysis on preoperative period. After surgery, others five patients started dialysis, resulting in 10 (6.7%) patients dialyzing 6 months after nephrectomy. Three patients of stage V migrated to stage IV within 6 months after the procedure. Four patients of stage V evolved to death due to sepsis.

**Table 2:** Postoperative complications data.

Clavien Grade	n(%)
I	121 (81.2)
II	12 (8.0)
IIIa	4 (2.6)
IIIb	2 (1.3)
IVa	3 (2.0)
IVb	3 (2.0)
V	4 (2.6)

On univariable analysis, higher preoperative chronic kidney stage (p = 0.02), Charlson comorbidity index ≥ 2 (p = 0.02), higher ASA score (p = 0.001), urgency due to sepsis (p = 0.01), kidney size ≥ 12 cm (p = 0.006), renal and perirenal abscess (p = 0.004 and 0.002 respectively) and visceral adhesion (p = 0.043) were associated with Clavien - Dindo score > 1 (Table-3). On multivariate analysis, higher ASA score (p = 0.01), urgency due to sepsis (p = 0.03), kidney size ≥ 12 cm (p = 0.04) and preoperative abscess (p = 0.04) remained significantly associated with Clavien - Dindo score > 1 (Table-4).

**Table 3:** Univariable analysis of risk factors for Clavien > 1 in nephrectomy for urolithiasis.

		Clavien 1 (n 121)	Clavien 2-5 (n 28)	p value
**Sex**				**0.83**
	Male	28 (23.1)	7 (25)	
	Female	93 (76.8)	21 (75)	
Age > 70y		5 (4.1)	2 (7.1)	0.49
BMI ≥ 30 (kg/m^2^)		24 (19.8)	5 (17.8)	1.00
**Preoperative CKD Stage**				**0.02**
	I	19 (15.7)	4 (14.2)	
	II	59 (48.7)	7 (25)	
	III	35 (28.9)	11 (39.2)	
	IV	1 (0.8)	2 (7.1)	
	V	7 (5.7)	4 (14.2)	
Staghorn		83 (68.5)	21 (75)	0.5
Charlson >2		37 (30.5)	15 (53.5)	0.02
Diabetes		17 (14.0)	4 (14.2)	0.97
Kidney Size ≥ 12cm		45 (37.2)	19 (67.8)	0.006
Urgency (Sepsis)		21 (17.4)	11 (39.2)	0.01
**Tomographic Findings**				
	Hydronephrosis	97 (80.1)	22 (78.5)	0.13
	Fat infiltration	83 (68.5)	26 (92.8)	0.02
	Renal abscess	36 (29.7)	17 (60.7)	0.004
	Perirenal abscess	20 (16.5)	13 (46.4)	0.002
	Pararenal abscess	15 (12.4)	7 (25)	0.14
	Fistula	9 (7.4)	4 (14.2)	0.27
	Adherence to the liver/spleen	33 (27.3)	14 (50)	0.04
	Adherence to the bowel	24 (19.8)	12 (42.8)	0.02
	Adherence to the muscle	30 (24.8)	8 (28.5)	0.81
**ASA**				**0.001**
	I	38 (31.4)	5 (17.8)	
	II	66 (54.5)	8 (28.5)	
	III	14 (11.5)	14 (50)	
	IV	3 (2.4)	1 (3.5)	

**BMI =** Body Mass Index; **CKD =** Chronic Kidney Disease; **CCI =** Charlson Comorbidity Index; **ASA =** American Society of Anesthesiologists.

**Table 4:** Multivariable logistic regression analyses predicting postoperative complications.

	OR (95% CI)	p-value
Preoperative CKD Stage	0.99 (0.98-1.01)	0.47
Charlson >2	0.84 (0.24-3.1)	0.84
ASA	2.31 (1.33-4.0)	0.01
Kidney Size ≥12cm	3.20 (1.32-7.5)	0.04
Urgency (Sepsis)	3.4 (1.27-11.96)	0.03
Fat Infiltration	3.96 (0.84-13.92)	0.36
Renal Abscess	3.34 (0.98-7.71)	0.05
Perirrenal Abscess	4.1 (0.71-9.84)	0.42
Abscess	3.86 (1.65-8.99)	0.04
Adherence to the Liver/Spleen	2.49 (0.91-5.71)	0.32
Adherence to the bowel	2.85 (0.88-6.76)	0.62

**OR =** Odds Ratio; CI: Confidence Interval; **CKD =** Chronic Kidney Disease; **CCI =** Charlson Comorbidity Index; **ASA =** American Society of Anesthesiologists

Dialysis was indicated in 3.4% (5 / 144) of patients after surgery. Higher preoperative chronic kidney stage (p = 0.002), Charlson > 2 (p = 0.005), higher ASA score (p = 0.005) and higher body mass index (BMI) (p = 0.03) were associated with postoperative dialysis on univariable analysis (Table-5). Patients who underwent surgery due to urgency due to sepsis needed longer hospital stay than elective surgery (12.9 ± 13.7 vs. 4.7 ± 5.1; p = < 0.001).

**Table 5:** Univariable analysis of risk factors for dialysis in nephrectomy for urolithiasis.

		Dialysis (%)		
		Yes	No	p-value
DMSA<20%		2 (40)	117 (84.1)	0.33
BMI ≥ 30 (kg/m^2^)		3 (60)	26 (18.7)	0.037
**Preoperative CKD**				0.002
	1	1 (20)	23 (16.5)	
	2	1 (20)	65 (46.7)	
	3	0	46 (33)	
	4	1 (20)	2 (1.4)	
	5	2 (40)	9 (6.4)	
Charlson > 2		5 (100)	47 (33.8)	0.005
**ASA**				0.005
	1	0	44 (31.6)	
	2	1 (20)	73 (52.5)	
	3	3 (60)	25 (17.9)	
	4	1 (20)	2 (1.4)	
Kidney size ≥12cm		3 (60)	61 (43.8)	0.65
**Tomographic Findings**				
	Hydronephrosis	1(20)	118 (84.8)	0.07
	Fat infiltration	1 (20)	108 (77.6)	0.19
	Renal abscess	0	53 (38.1)	1
	Perirenal abscess	2 (40)	31 (22.3)	0.31
	Pararenal abscess	0	22 (15.8)	1
	Fistula	0	13 (9.3)	1
	Adherence to the liver/spleen	1 (20)	46 (33)	1
	Adherence to the bowel	1 (20)	35 (25.1)	1
	Adherence to the muscle	1 (20)	37 (26.6)	0.32

**CKD =** Chronic Kidney Disease; **CCI =** Charlson Comorbidity Index; **ASA =** American Society of Anesthesiologists

## DISCUSSION

Urolithiasis is the leading cause of nephrectomy for benign conditions (8), necessary in case of severe urinary infection or chronic pain in a renal unit with a poor function (9). Nephrectomy performed outside Gerota's fascia is our preferred technique when is necessary, mimicking radical nephrectomy for kidney cancer. This way, the surgeon may approach the renal hilum far from the most intense inflammatory process caused by the stone itself and stressed by infection (10). Previous infections can lead to dense adhesions, especially in the perinephritic region. It is usual for the surgeon to have difficulties in the renal hilum due to the presence of bulky adenopathies, fat infiltration, fibroses and adhesions to near structures, like bowel and pancreas, resulting in inadvertent injuries. These particularities may result in higher complication rates. In a recent study, Zelhof et al. (2) reviewed 1093 cases of nephrectomy for benign diseases and showed that patients with stone disease had higher complication rate (23.9%) comparing to chronic pyelonephritis (13.2%) and non - functioning kidney (9.1%). The investigators also showed that in comparison with radical nephrectomy (T1 renal tumors only), procedures for benign disease had higher complication (11.9% vs. 10.0%), conversion (5.9% vs. 3.3%) and transfusion rates (4.8 vs. 2.8%). Tepeler et al. (11) compared patients submitted to retroperitoneoscopic nephrectomy for renal stone and other benign disease and found that the peri - and postoperative complications rates were higher in the stone group. Considering Clavien - Dindo score > 1, our study evidenced a complication rate of 19.3%. Vascular injury is the most common major injury during laparoscopic surgery. The literature evidences that vascular injury rates in these patients is around 0.8 – 2.6% (12, 13). In our study, there were five (3.3%) cases of vascular injury. Vena cava was involved in three cases. It can be justified by the proximity of the vena cava to the renal hilum on the right side associated with inflammatory conditions and fibrosis. In a study comparing laparoscopic versus open nephrectomy for inflammatory diseases, the rate of pleural injury was 12.3% (14). In the present study, two patients (1.3%) had pleural injury during the nephrectomy.

Laparoscopic nephrectomy for urolithiasis and inflammatory conditions has generally been associated with a high open conversion rate. A study with 62 laparoscopic simple nephrectomies for non malignancy causes showed that conversion to open surgery was necessary in seven cases (7.2%) because it proved impossible to dissect the renal hilum owing to xanthogranulomatous pyelonephritis (n = 4) or major associated lesions (n = 3) (10). Other series with 50 patients submitted to laparoscopic nephrectomy for inflammatory conditions, conversion was verified in 14 (28.0%) cases, owing to severe adhesions and fibrosis (7). These conversion rates appear to be higher when compared to radical nephrectomy. Permpongkosol et al. reviewed their complications of 2775 urological laparoscopic procedures and found that open conversion rate was doubled for laparoscopic simple nephrectomy versus laparoscopic radical nephrectomy (5.9% vs. 2.9%, respectively) (15). We evaluated 83 cases of laparoscopic nephrectomies and our conversion rate (19.2%) has remained in the patterns of the current literature, but still high compared to radical nephrectomy.

There are few evidences in medical literature establishing predictive factors for complications after nephrectomy for urolithiasis. A British study with its first 100 cases with laparoscopic nephrectomy, including 12 cases with stones, evidenced inflammatory conditions (xanthogranulomatous pyelonephritis and pyonephrosis) and previous renal surgery as risk factors for complications (16). Manohar et al. evaluated 84 cases of laparoscopic nephrectomy due to inflammatory conditions and showed that kidney size > 10 cm and presence of hilar lymphadenopathy were predictors of a higher complication rate (14). In other series with laparoscopic urological surgeries, high comorbidity index had a marginal association with the incidence of complications (p = 0.06) and low ASA score had a protector factor for complications (p = 0.04) (17). In the current study after multivariable analysis, higher ASA score (p = 0.01), urgency due to sepsis (p = 0.03), kidney size ≥ 12 cm (p = 0.04) and preoperative abscess (p = 0.04) were associated with Clavien - Dindo score > 1. The high ASA score as a risk factor can be justified by the considerable number of patients with severe urinary tract infection and sepsis (21.4%). Until now, our study is the only one that analyzes the risk factor for complications exclusively in nephrectomy due to lithiasis.

Preoperative radiological evaluation with computed tomography plays an important role in the surgical planning of nephrectomy for urinary stones. The association with urinary infection can result in anatomic changes in urinary tract and near structures. Some computed tomography findings could anticipate the complexity and prepare the surgeon for the renal approach. In the present study, we have categorized some findings in an attempt to find risk factors for complications. Sixty four patients had a kidney size ≥ 12 cm. Hydronephrosis was the most common finding (79.8%), followed by fat stranding (73.1%), renal, perirenal and pararenal abscesses (35.5%, 22.1% and 14.7%, respectively), adherences to the liver / spleen, muscle and bowel (31.5%, 24.1%, 25.5%, respectively) and fistula (8.7%). Only kidney size ≥ 12 cm (p = 0.04) and preoperative abscess (p = 0.04) resulted as significant predictive factor of the development of postoperative complications.

Radical nephrectomy is an independent risk factor for decreased renal function (18). It is reported that the acute renal failure rate after nephrectomy is about 0.4% (19). A multicenter study, including 2454 patients showed that age > 58 years, preoperative serum creatinine > 1.03 mg / mL, and EGFR < 73 mL / min/1.73 m^2^ had a higher probability of developing post - nephrectomy chronic renal insufficiency (20). In the present study, 10 (6.7%) patients needed dialysis up to 6 months after nephrectomy and one of them was submitted to renal transplantation during this period. These patients need a close follow-up to assess renal function after surgery.

Our study has some limitations. Surgical approach was biased by surgeon and patient preferences. As far as we know, this is the first report to look for predictive factors for complications in nephrectomy due to stone disease. A prospective multi institutional study with a large number of patients is desired to confirm our data.

In conclusion, nephrectomy for stone disease presents high complication rates and deserves special attention by surgeons. We identified that higher ASA score, urgency due to sepsis, kidney size ≥ 12 cm and preoperative abscess were associated with Clavien - Dindo score > 1. Under these conditions, we suggest a thorough preoperative evaluation with computed tomography and observing the comorbidities involved, in order to identify the cases with a greater probability of complications, thus attracting more attention of the surgeon. Furthermore, predictors for postoperative dialysis were higher chronic kidney stage, higher Charlson comorbidity index, higher ASA score and higher BMI.
